# The value of cardiac magnetic resonance post-contrast T1 mapping in improving the evaluation of myocardial infarction

**DOI:** 10.3389/fcvm.2023.1238451

**Published:** 2023-10-16

**Authors:** Chunlin Xiang, Hongyan Zhang, Haojie Li, Xiaoyue Zhou, Lu Huang, Liming Xia

**Affiliations:** ^1^ Department of Radiology, Tongji Hospital, Tongji Medical College, Huazhong University of Science and Technology, Wuhan, China; ^2^ Department of Gynecology, Tongji Hospital, Tongji Medical College, Huazhong University of Science and Technology, Wuhan, China; ^3^ Siemens Healthineers Digital Technology (Shanghai) Co., Ltd., Shanghai, China

**Keywords:** cardiac magnetic resonance, myocardial infarction, T1 mapping, subendocardial scar, late gadolinium enhancement

## Abstract

**Objective:**

To explore the additional value of cardiac magnetic resonance (CMR) post-contrast T1 mapping in the detection of myocardial infarction, compared with late gadolinium enhancement (LGE).

**Materials and methods:**

A CMR database of consecutive patients with myocardial infarction was retrospectively analyzed. All patients were scanned at 3 T magnetic resonance; they underwent conventional CMR (including LGE) and post-contrast T1 mapping imaging. Two radiologists interpreted the CMR images using a 16-segment model. The first interpretation included only LGE images. After 30 days, the same radiologists performed a second analysis of random LGE images, with the addition of post-contrast T1 mapping images. Images were analyzed to diagnose myocardial scars, and the transmural extent of each scar was visually evaluated. Diagnoses retained after LGE were compared with diagnoses retained after the addition of post-contrast T1 mapping.

**Results:**

In total, 80 patients (1,280 myocardial segments) were included in the final analysis. After the addition of post-contrast T1 mapping, eight previously unidentified subendocardial scars were detected. Compared with LGE images, the percentage of infarcted segments was higher after the addition of post-contrast T1 mapping images (21.7% vs. 22.3%, *P* = 0.008), the percentage of uncertain segments was lower after the addition of post-contrast T1 mapping (0.8% vs. 0.1%, *P* = 0.004), and the percentage of uncertain transmural extent of scarring was lower after the addition of post-contrast T1 mapping (0.9% vs. 0.1%, *P* = 0.001).

**Conclusion:**

The addition of post-contrast T1 mapping after LGE helps to improve the detection of myocardial infarction, as well as the assessment of the transmural extent of scarring.

## Introduction

Cardiovascular disease is the greatest threat to human health worldwide, and myocardial infarction is the leading cause of death (1, 2). The burden of myocardial infarction is growing because of the increasing prevalence of myocardial infarction risk factors, as well as high morbidity and mortality rates associated with myocardial infarction (3, 4). Therefore, health prevention, accurate diagnosis, and timely treatment of myocardial infarction are urgent challenges. Currently, despite improvements in the treatment of myocardial infarction, such as percutaneous coronary intervention (PCI), patients continue to experience high risks of heart failure and death (5). Therefore, assessments of myocardial infarction scar detection, size, and transmural extent of scarring are needed to guide treatment decisions and predict long-term prognoses.

Over the past two decades, rapid developments in magnetic resonance technology have helped cardiac magnetic resonance (CMR) to become an essential method for evaluation of cardiovascular diseases. CMR is a non-invasive imaging method with that exhibits high resolution, good contrast, and no ionizing radiation; it can be used for multi-sequence imaging (6).

CMR can also characterize histopathological changes in the myocardium, such as edema, necrosis, and fibrosis. Late gadolinium enhancement (LGE) is an essential method for non-invasive examinations of myocardial pathology; it has been widely used to assess myocardial infarction. LGE uses an inversion recovery (IR) sequence to attenuate the signal of normal myocardium; it can distinguish a bright myocardial scar from normal black myocardium. Ischemia-induced myocardial infarction initially affects subendocardial fibers of the myocardium, then gradually extends to the epicardium through the ventricular wall (i.e., the wavefront phenomenon) (7).

In LGE images, the high signal of left ventricular blood may lead to poor contrast between a subendocardial scar and adjacent blood. This may hinder identification of the subendocardial scar or reduce diagnostic confidence (8). Because a subendocardial scar produces only a slight change in ventricular wall motion, it may be missed in the cine sequence (9). In some instances, poor contrast leads to a lack of clarity regarding the exact boundary between the scar and adjacent blood, affecting assessment of the transmural scar and extent of endocardial involvement.

In recent years, quantitative mapping technology has played an increasingly important role in the evaluation of cardiovascular diseases. Myocardial mapping technology provides specific parameters of the myocardium (T1, T2, T2 *, and extracellular volume), which can reflect changes in myocardial tissue composition (10). Post-contrast T1 mapping uses the modified Look-Locker inversion recovery (MOLLI) sequence to synthesize a color map, which can visually display differences in T1 values of myocardial tissue and facilitate the quantification of T1 values (11). The rate of gadolinium contrast agent removal from myocardial infarction scars is slower than the rate of removal from normal myocardium, thereby reducing the T1 value of the scar. Generally, 10–20 min after injection of contrast agent, the scar exhibits a high signal on LGE; on T1 mapping, it exhibits a lower T1 value than normal myocardium.

Post-contrast T1 mapping has shown promise in detecting myocardial scars and quantifying the extent of scarring in previous studies (12, 13). However, considering the limitations of LGE in diagnosing subendocardial scars, it is unclear whether post-contrast T1 mapping can provide additional diagnostic value. In this study, we retrospectively analyzed CMR data from patients with confirmed myocardial infarction to explore the value of post-contrast T1 mapping in the evaluation of myocardial infarction.

## Materials and methods

### Study population

**Figure 1:**
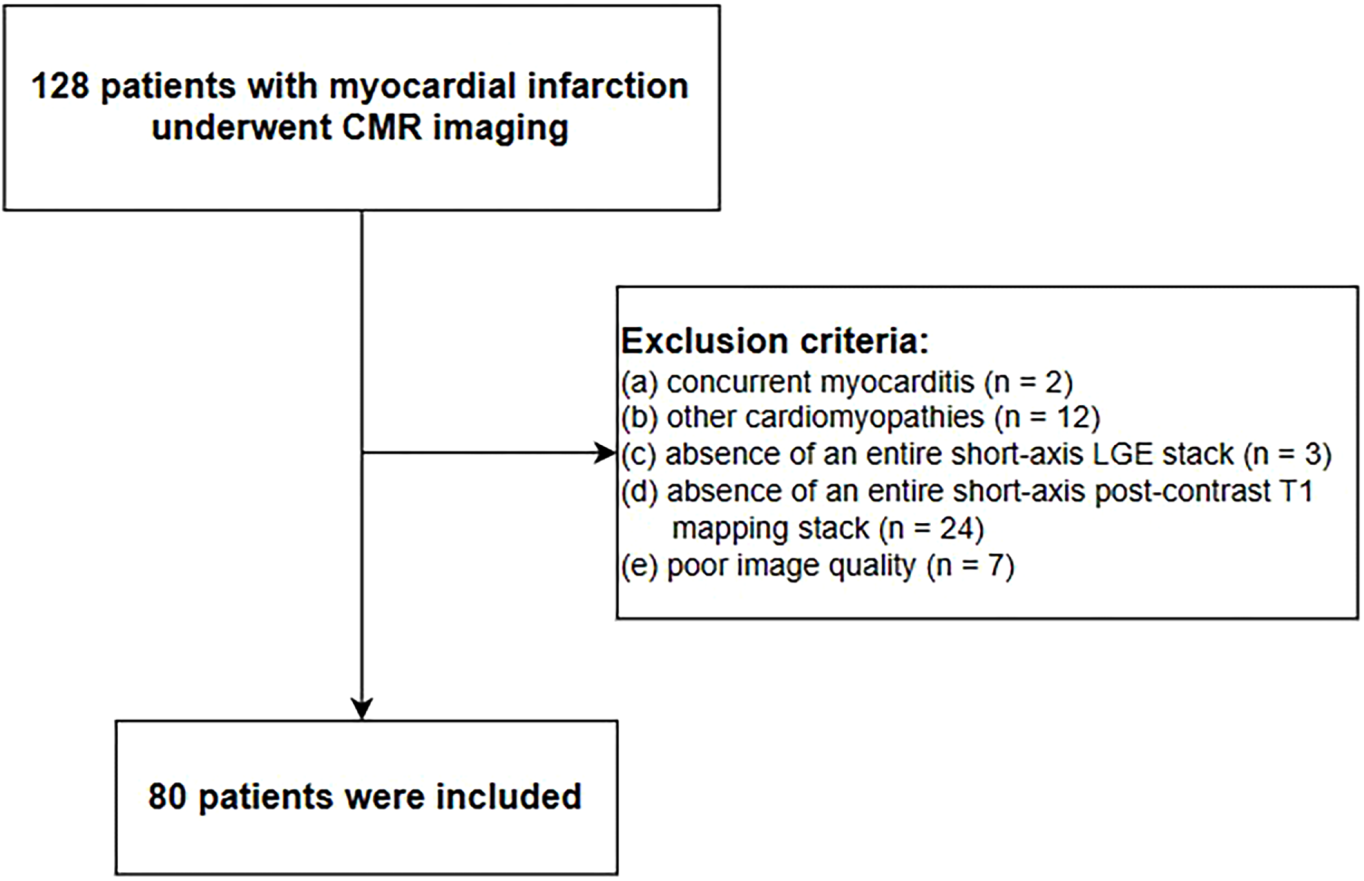
Flowchart of patient selection.

The institutional ethics review committee at our institution approved this study and waived the requirement for informed consent. In total, 128 consecutive patients with myocardial infarction who underwent CMR imaging in our department from June 2019 to February 2022 were included in this study. The clinical diagnostic criteria for myocardial infarction were based on the "Fourth Universal Definition of Myocardial Infarction (2018)" (14); specifically, myocardial infarction was defined as the detection of an elevated cardiac troponin I value above the 99th percentile upper reference limit, accompanied by clinical or imaging evidence of myocardial ischemia. In this study, high-sensitivity cardiac troponin I (hs-cTnI) > 34.2 pg/ml was regarded as an elevated cardiac troponin I value. Evidence of myocardial ischemia was primarily assessed by symptoms of ischemia, ischemic changes on electrocardiography, presence of Q waves, and characteristic imaging evidence. Forty-eight patients were excluded from this study based on the following exclusion criteria: concurrent myocarditis, other cardiomyopathies, absence of an entire short-axis LGE stack, absence of an entire short-axis post-contrast T1 mapping stack, or poor image quality. Ultimately, 80 patients were included in this study. The flowchart of patient selection is shown in Figure 1.

### CMR acquisition

All patients underwent CMR on a 3-T magnetic resonance scanner (MAGNETOM Skyra, Siemens Healthineers, Erlangen, Germany) with an 18-channel body coil and a 32-channel spine coil. LGE was scanned 10 min after the injection of 0.15 mmol/kg gadolinium contrast agent, post-contrast T1 mapping was scanned immediately after the LGE scan, and all LGE and post-contrast T1 mapping scans were performed 10–20 min after contrast agent injection. The stack of 8–12 short-axis slices covered the left ventricle from base to apex. Subsequently, images were scanned with a breath-hold of 7–14 s (depending on the patient's heart rate) during the diastolic period.

LGE was performed using a segmented phase-sensitive inversion recovery (PSIR) sequence. The number of k segments per one cardiac cycle was 28, and 10 cardiac cycles were necessary for one slice. The main scanning parameters were as follows: repetition time = 3.2 ms, echo time = 1.2 ms, matrix = 224 × 150, flip angle = 55°, field of view = 360 × 320 mm^2^, slice thickness = 8 mm, and bandwidth = 770 Hz. The selection of the inversion time (TI) value was based on a TI-scout sequence using multiple inversion times. The optimal TI value was adapted to null the signal of normal myocardium, typically in the range of 280 to 330 ms.

Post-contrast T1 mapping was performed immediately after LGE using the MOLLI sequence with the acquisition protocol 4b(1b)3b(1b)2b, where b represents heartbeat. The main scanning parameters were as follows: repetition time = 2.84 ms, echo time = 1.2 ms, matrix = 256 × 172, flip angle = 35°, field of view = 360 × 320 mm^2^, slice thickness = 8 mm, and bandwidth = 1,085 Hz; three inversion pulses were used (initial inversion delay of 117 ms, with two increments of 80 ms). The acquired original images were fitted inline by motion correction and a non-linear least-squares curve to generate a pixel-wise colored T1 map.

### Image analysis

LGE and post-contrast T1 mapping images were divided into 16 myocardial segments (excluding the apex), in accordance with the American Heart Association segmentation model (15). Two radiologists (with 5 and 10 years of CMR diagnosis experience, respectively) independently interpreted the CMR images in random order. The first interpretation included LGE images: magnitude and PSIR images, both viewed in a side-by-side manner. After 30 days, the same radiologists performed a second analysis of random LGE images, with the addition of post-contrast T1 mapping images. During the second interpretation, both radiologists were blinded to the results of the first interpretation. LGE and post-contrast T1 mapping images were analyzed to identify myocardial scars. Compared with remote normal myocardium, each myocardial scar was defined as a region five standard deviations higher than on LGE or two standard deviations lower on post-contrast T1 mapping. Images were interpreted in terms of myocardial segments, and results were classified into three categories: infarcted, uncertain, and negative. Infarcted and negative segments were defined as segments for which the radiologist was confident about the interpretation; uncertain segments were defined as segments for which the interpretation was unclear. In each infarcted segment, the maximum transmural scar was visually evaluated using a 4-point scale (1 = 1%–25%, 2 = 26%–50%, 3 = 51%–75%, and 4 = 76%–100%). For scars with uncertain transmural extent, the number of involved segments was quantified as a percentage of the total number of segments. Scars were considered transmural if more than 75% penetration through the cardiac wall. All data were randomly analyzed twice to evaluate intraobserver and interobserver agreement for the two radiologists.

### Statistical analysis

Continuous quantitative data were expressed as means ± standard deviations or medians (interquartile ranges); categorical data were expressed as frequencies (percentages). The Shapiro-Wilk test was used to assess whether continuous quantitative data conformed to a normal distribution. Continuous variables were analyzed using independent samples *t*-tests (normally distributed data) or the Mann-Whitney *U* test (non-normally distributed data). Categorical variables were analyzed using the McNemar chi-squared test when for frequencies ≥ 5 and Fisher's exact test for frequencies < 5. Interobserver and intraobserver agreements were analyzed using Cohen's kappa coefficient (excellent, kappa ≥ 0.75; moderate, 0.4 ≤ kappa < 0.75; and poor, kappa < 0.4). SPSS 21.0 software (IBM Corporation, Armonk, NY, USA) was used for statistical analysis. *p*-values < 0.05 were considered statistically significant.

## Results

### Patient characteristics

For patients with acute or subacute myocardial infarction, CMR was performed 3 days after coronary angiography (CAG) or PCI; for patients with chronic myocardial infarction, CMR was performed during the 3-month follow-up period after CAG or PCI. Overall, 80 patients (1,280 myocardial segments) were included in this study; their clinical characteristics are shown in Table 1. The mean age was 53 ± 12 years, and 68 (85%) of the patients were men. The median hs-cTnI peak was 6,315.9 pg/ml (range, 110.7–160,000 pg/ml). Electrocardiography showed ST-segment elevation in 50 patients (62.5%) and Q waves in 38 patients (47.5%). CAG was performed in 70 patients, of whom 52 (74.3%) had at least one coronary artery stenosis (≥ 50%) and 46 (57.5%) had undergone PCI.

**Table 1:** Characteristics of patients with myocardial infarction.

Clinical information	Total
Male	68 (85%)
Age (years)	53 ± 12
Height (cm)	168 ± 6
Weight (kg)	71 ± 9
Hypertension	50 (62.5%)
Dyslipidemia	36 (45%)
Diabetes	18 (22.5%)
Heart rate (beats/min)	71 ± 9
LVEF (%)	52 ± 15
hs-cTnI (pg/ml)	6,315 (1,325.2, 22,985)
ST-segment elevation	50 (62.5%)
Q waves	38 (47.5%)
PCI	46 (57.5%)
CAG (*n* = 70)	
LM	2 (2.9%)
LAD	42 (60%)
LCX	32 (45.7%)
RCA	32 (45.7%)

Values are shown as *n* (%) or median (interquartile range).

LVEF, left ventricular ejection fraction; PCI, percutaneous coronary intervention; hs-cTnI, high-sensitivity cardiac troponin I; LM, left main coronary artery; LAD, circumflex branch of left coronary artery; LCX, left anterior descending coronary artery; RCA, right coronary artery; CAG, coronary angiography.

### Comparison of LGE alone and the addition of post-contrast T1 mapping

**Figure 2:**
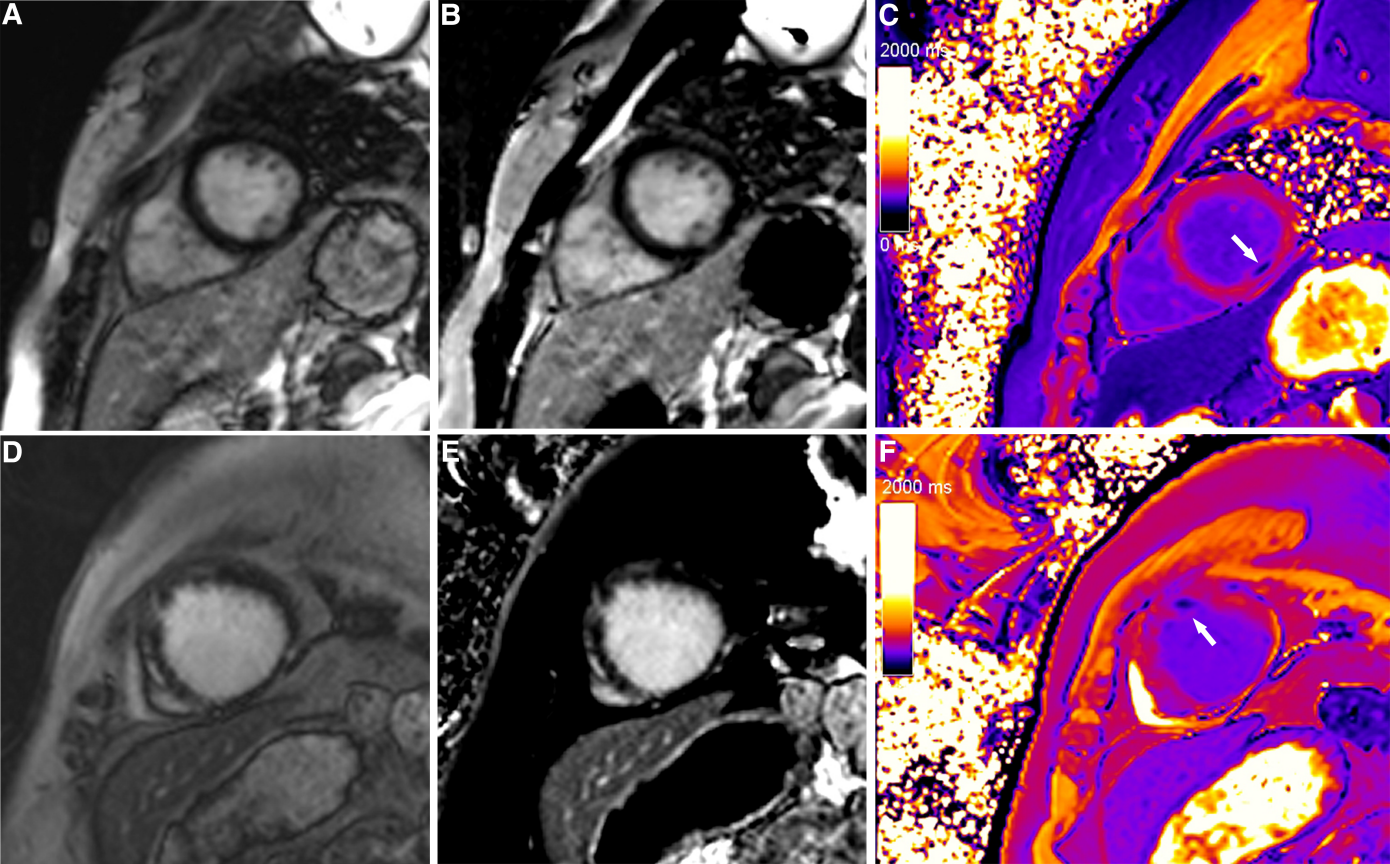
Typical cases in which post-contrast T1 mapping improved the detection of infarcted segments. A focal subendocardial scar was missed on magnitude (A, D) and PSIR (B, E), and an additional scar (white arrow) was identified on post-contrast T1 mapping (C, F).

**Figure 3:**
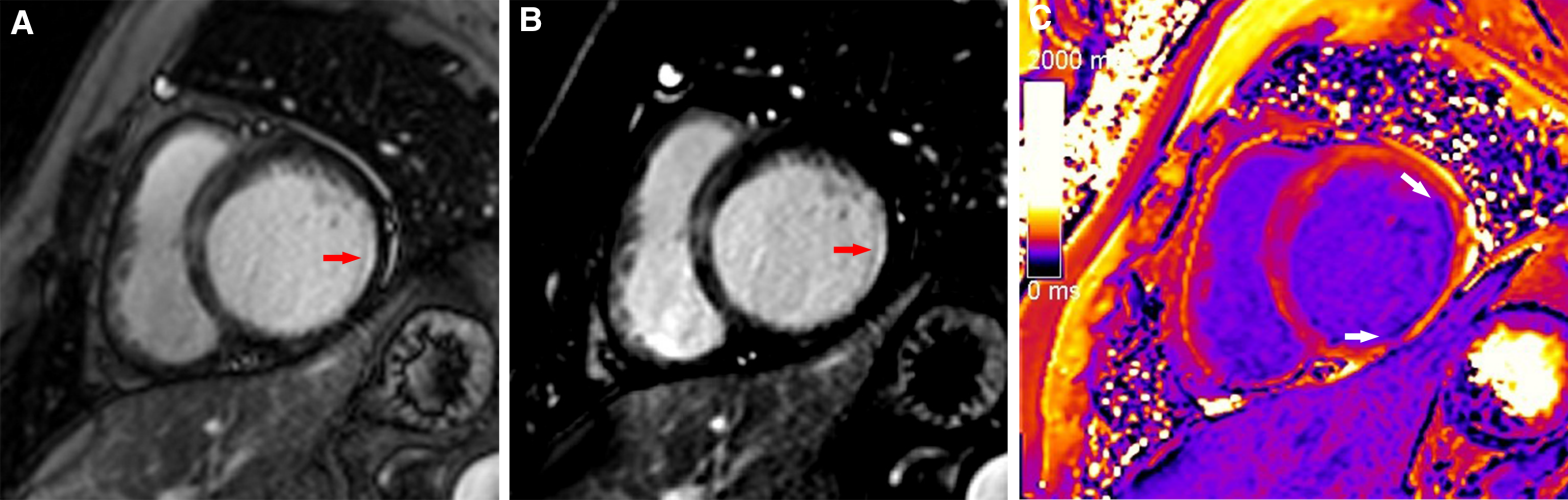
A typical case in which post-contrast T1 mapping improved the detection of subendocardial scarring. A subendocardial scar (red arrow) could be observed on magnitude (A) and PSIR (B) in the lateral wall of the base. The scar visibility was poor because it was adjacent to bright blood, making it challenging to evaluate the extent of subendocardial scarring. Greater extent of subendocardial scarring (white arrows) could be observed on post-contrast T1 mapping (C).

**Figure 4:**
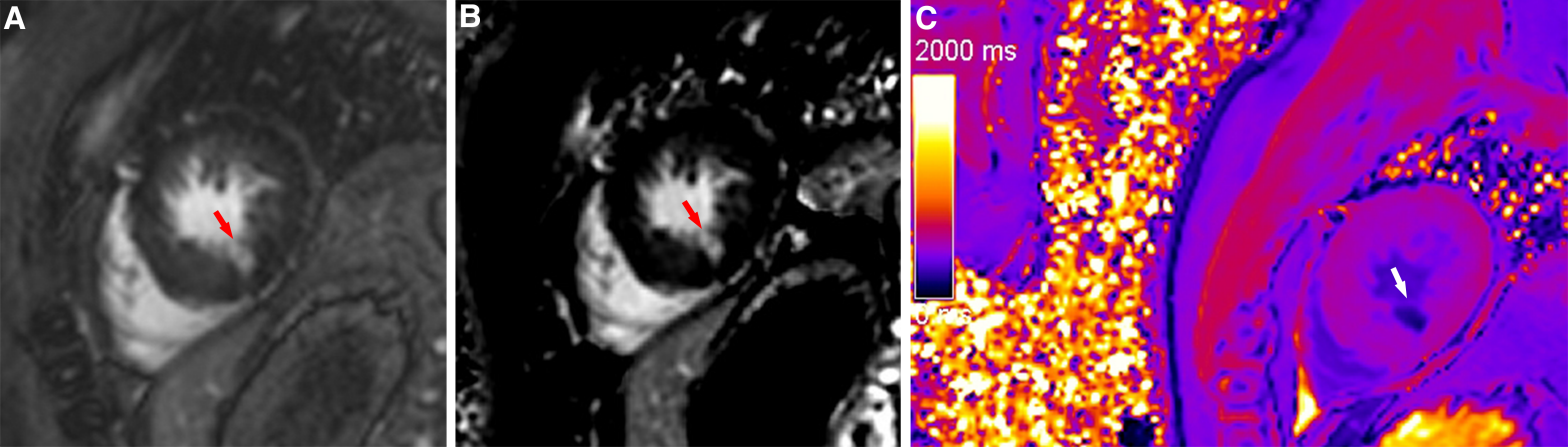
A typical case in which post-contrast T1 mapping reduced the number of uncertain infarcted segments. An ambiguous focal scar (red arrow) could be observed on magnitude (A) and PSIR (B) in the inferior subendocardium of the apex, but the observer's diagnostic confidence was low. The observer's diagnostic confidence in the scar (white arrow) was improved on post-contrast T1 mapping (C); thus, the affected segment was classified as an infarcted segment.

**Figure 5:**
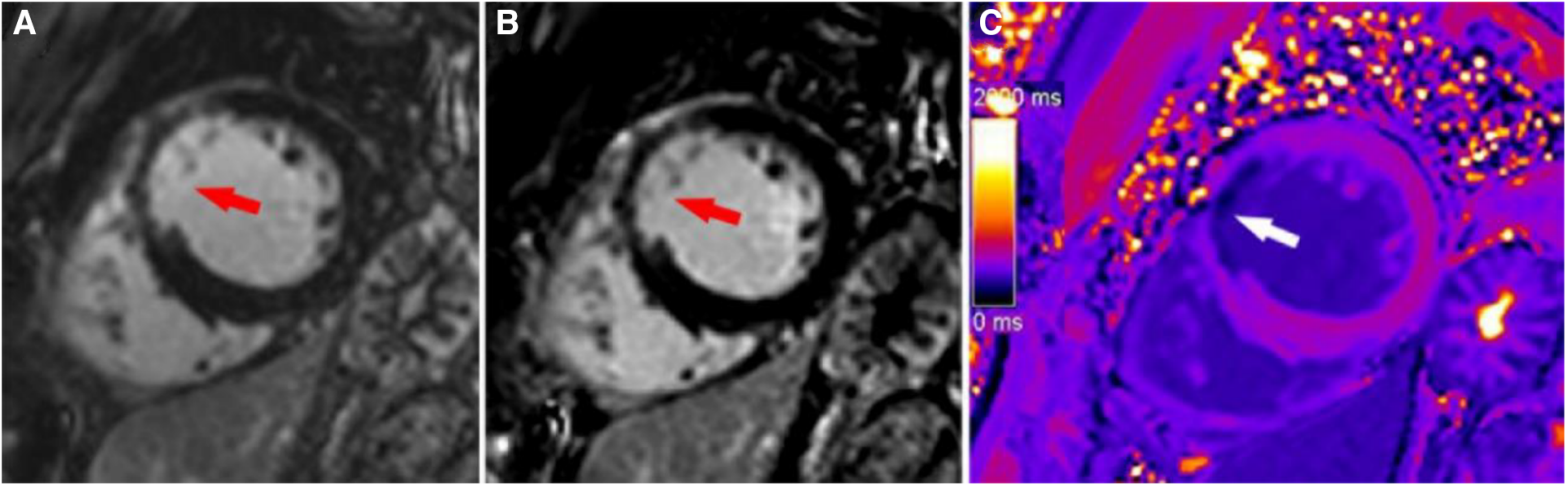
A typical case in which post-contrast T1 mapping improved transmural scar detection. A high-signal scar (red arrow) was observed on magnitude (A) and PSIR (B); however, poor contrast between the scar and blood hindered accurate delineation of the endocardial border and influenced assessment of the transmural extent of scarring. Post-contrast T1 mapping (C) provided greater border clarity (white arrow), improving the accuracy of assessment regarding the transmural extent of scarring.

After the addition of post-contrast T1 mapping, eight previously unidentified scars were detected. Among these eight segments, four (50%) were located in the basal segment, one (12.5%) was located in the middle segment, and three (37.5%) were located in the apical segment. The percentage of infarcted segments was higher after post-contrast T1 mapping than after LGE alone (22.3% vs. 21.7%, *P* = 0.008); the percentages of uncertain segments and uncertain transmural extent of scarring were both lower after the addition of post-contrast T1 mapping (0.1% vs. 0.8%, *P* = 0.004; 0.1% vs. 0.9%, *P* = 0.001). A comparison of LGE alone and the addition of post-contrast T1 mapping is shown in Table 2; typical cases are depicted in Figures 2–5. Inter- and intraobserver agreements were excellent for both LGE (kappa = 0.85 and kappa = 0.90, respectively) and the addition of post-contrast T1 mapping (kappa = 0.88 and kappa = 0.95, respectively).

**Table 2:** Comparison of LGE alone and the addition of post-contrast T1 mapping.

	LGE alone	LGE and post-contrast T1 mapping	*P-*value
Infarcted segments	278 (21.7%)	286 (22.3%)	0.008
Uncertain segments	10 (0.8%)	1 (0.1%)	0.004
Negative segments	992 (77.5%)	993 (77.6%)	1.000
Transmural scar	110 (8.6%)	112 (8.8%)	0.5
Subendocardial scar	156 (12.1%)	173 (13.5%)	<0.001
Uncertain transmural extent	12 (0.9%)	1 (0.1%)	0.001

LGE, late gadolinium enhancement.

## Discussion

This study retrospectively evaluated the additional value of CMR post-contrast T1 mapping in the detection of myocardial infarction. The results showed that the addition of post-contrast T1 mapping to LGE can improve the detection of infarcted segments, reduce the percentage of uncertain myocardial segments, and reduce the percentage of uncertain extent of transmural scarring.

When LGE nulls normal myocardium through the IR sequence, subendocardial scars and blood both exhibit high signals, which may cause scars to be missed because of the lack of contrast. Additionally, the selection of an appropriate TI value and presence of cardiac motion artifacts during LGE imaging may affect image quality and scar detection. The addition of post-contrast T1 mapping can resolve some limitations of LGE. The principle of T1 mapping is that myocardial tissue characteristics are represented by specific T1 values. T1 maps constitute pixel-wise, color-coded quantitative maps that can directly reflect subtle changes in T1 values and provide additional diagnostic information for myocardial assessment (16, 17).

Because of the shortening effect of the gadolinium contrast agent, the T1 value of a scar is lowest 10–20 min after contrast agent injection, and the scar is visible on post-contrast T1 mapping. Importantly, post-contrast T1 mapping avoids dependence on the selection of an appropriate TI value. Compared with LGE using segmented PSIR, T1 mapping with motion correction can reduce motion artifacts in some images with poor breath holding, thereby facilitating evaluation of scarring.

The results of the present study will allow clinicians to acquire additional information regarding patient prognosis, which could influence patient management and treatment strategies. Because scar size is closely associated with many adverse outcomes (18, 19) and even small myocardial scars have a substantial impact on prognosis (20, 21), accurate detection and size delineation of subendocardial scars is important for patients with a history of myocardial infarction.

To improve contrast between scars and blood, researchers have used various preparation pulses that produce dark or gray blood LGE by inhibiting the signal of left ventricular blood (22–27); these approaches improve subendocardial scar detection. However, a potential disadvantage of the dark or gray blood sequence is reduced contrast between the scar and normal myocardium, which can affect the detection of small scars within normal myocardium. Preparation pulses can also be a major source of image artifacts and affect overall image quality. Finally, the dark or gray blood sequence is technically challenging to perform; it requires sequence optimization and additional technical training. Because most preparation pulse sequences are in early stages of exploration, they are not widely used in clinical practice. However, since the first publication about T1 mapping in 1988 (28), T1 mapping technology has matured to achieve high accuracy, repeatability, and consistency (29, 30). Therefore, T1 mapping is increasingly utilized in routine clinical practice.

Holtackers et al. (31) found that setting the TI value of blood to null in the standard PSIR pulse sequence for LGE imaging increases contrast between the scar and blood, improving myocardial scar detection without additional pulse preparation. However, an accurate TI value must be selected to ensure attenuation of the blood signal. Because of contrast agent outflow, the time window for LGE imaging is limited, and it is difficult to select an appropriate TI value. Therefore, the acquisition of a TI-scout sequence often prolongs the imaging time. Additionally, the signal difference between the scar and normal myocardium may be decreased when the blood signal is nulling, which may affect the detection of small scars or fibrotic lesions.

Some challenges remain in the imaging-based detection of myocardial infarction. This type of imaging constitutes an area of research in which a new method can improve decision making, risk stratification, and management. Ischemia-induced myocardial necrosis initially affects subendocardial fibers, then gradually progresses from the subendocardium to the epicardium. Therefore, accurate assessment of subendocardial scars requires attention, and the use of multiple sequences is essential. In our institution, T1 mapping has become a routine CMR scan. Considering the additional diagnostic value of post-contrast T1 mapping, we believe that the addition of 3–4 min of scanning time for patients with myocardial infarction is acceptable. We also recommend careful consideration of the diagnostic value of post-contrast T1 mapping among institutions that have implemented post-contrast T1 mapping scans.

This study had several limitations. First, the scars identified by post-contrast T1 mapping and LGE were not pathologically confirmed. To reduce false positives, this study only included patients with clinically diagnosed myocardial infarction; ischemia-induced myocardial infarction scars initially affect subendocardial tissue. In this study, all additional myocardial infarction scars identified after the addition of post-contrast T1 mapping originated from subendocardial tissue. Second, post-contrast T1 mapping may produce less common artifacts, such as errors related to blood-derived partial volume contamination, which must be carefully assessed. Third, extracellular volume maps were not included in this study, and their value should be explored in future research. Finally, this study only considered scars in patients diagnosed with myocardial infarction. Post-contrast T1 mapping may be useful in the diagnosis of scars caused by other diseases, such as hypertrophic cardiomyopathy, sarcoidosis, or myocarditis. Therefore, further analyses are needed concerning the clinical role of post-contrast T1 mapping in different populations and diseases.

In patients with myocardial infarction, the addition of post-contrast T1 mapping after LGE helps to improve the detection of myocardial infarction, as well as the assessment of the transmural extent of scarring.

## Data Availability

The original contributions presented in the study are included in the article/supplementary materials, further inquiries can be directed to the corresponding author/s.
